# Toxicity of Tungsten Carbide and Cobalt-Doped Tungsten Carbide Nanoparticles in Mammalian Cells *in Vitro*

**DOI:** 10.1289/ehp.0800121

**Published:** 2008-12-01

**Authors:** Susanne Bastian, Wibke Busch, Dana Kühnel, Armin Springer, Tobias Meißner, Roland Holke, Stefan Scholz, Maria Iwe, Wolfgang Pompe, Michael Gelinsky, Annegret Potthoff, Volkmar Richter, Chrysanthy Ikonomidou, Kristin Schirmer

**Affiliations:** 1 Department of Pediatric Neurology, University Children’s Hospital Carl Gustav Carus, University of Technology Dresden, Dresden, Germany;; 2 Department of Cell Toxicology, UFZ-Helmholtz Centre for Environmental Research, Leipzig, Germany;; 3 Max Bergmann Center of Biomaterials, Institute of Materials Science, University of Technology Dresden, Dresden, Germany;; 4 Fraunhofer Institute for Ceramic Technologies and Systems, Dresden, Germany;; 5 Eawag, Swiss Federal Institute of Aquatic Science and Technology, Dübendorf, Switzerland

**Keywords:** cellular uptake, cobalt doping, cobalt salt, human cell cultures, *in vitro*, nanoparticle behavior, toxicity, tungsten carbide nanoparticles

## Abstract

**Background:**

Tungsten carbide nanoparticles are being explored for their use in the manufacture of hard metals. To develop nanoparticles for broad applications, potential risks to human health and the environment should be evaluated and taken into consideration.

**Objective:**

We aimed to assess the toxicity of well-characterized tungsten carbide (WC) and cobaltdoped tungsten carbide (WC-Co) nanoparticle suspensions in an array of mammalian cells.

**Methods:**

We examined acute toxicity of WC and of WC-Co (10% weight content Co) nanoparticles in different human cell lines (lung, skin, and colon) as well as in rat neuronal and glial cells (i.e., primary neuronal and astroglial cultures and the oligodendro cyte precursor cell line OLN-93). Furthermore, using electron microscopy, we assessed whether nanoparticles can be taken up by living cells. We chose these *in vitro* systems in order to evaluate for potential toxicity of the nanoparticles in different mammalian organs (i.e., lung, skin, intestine, and brain).

**Results:**

Chemical–physical characterization confirmed that WC as well as WC-Co nanoparticles with a mean particle size of 145 nm form stable suspensions in serum-containing cell culture media. WC nanoparticles were not acutely toxic to the studied cell lines. However, cytotoxicity became apparent when particles were doped with Co. The most sensitive were astrocytes and colon epithelial cells. Cytotoxicity of WC-Co nanoparticles was higher than expected based on the ionic Co content of the particles. Analysis by electron microscopy demonstrated presence of WC nanoparticles within mammalian cells.

**Conclusions:**

Our findings demonstrate that doping of WC nanoparticles with Co markedly increases their cytotoxic effect and that the presence of WC-Co in particulate form is essential to elicit this combinatorial effect.

Nanotoxicology is an emerging field of research at the intersection of material science, medicine, and toxicology. The ultimate characteristic of nanomaterials is their size, which can modify the physicochemical properties of the material, enable increased uptake and interaction with biological tissues, and generate adverse biological effects in living cells that would not be possible with the same material in larger or soluble form. Smaller particle size leads to increased surface area and allows for a greater proportion of atoms or molecules to be displayed on the surface. Clinical and experimental studies indicate that a small size, a large surface area, and the ability to generate reactive oxygen species (ROS) contribute to the potential of nanoparticles to induce cell injury (Colvin 2003; Nel et al. 2006; Oberdörster et al. 2005).

Most toxicology data for engineered nanomaterials are derived from inhalation studies concentrating on lung injury and assessment of inflammatory parameters. Uptake of metal oxide nanoparticles in lung cells has been demonstrated *in vivo* as well as in different cell culture systems (Geiser et al. 2008; Limbach et al. 2005; Stearns et al. 2001). Toxic effects in human lung cells depend on particle composition and size and related reactivity (Brunner et al. 2006; Duffin et al. 2007; Limbach et al. 2005, 2007). So-called nano effects, meaning differing effects of nanomaterials compared with bulk materials of the same chemical composition, have been observed, with nanomaterials being more toxic in regard to reduction of cell viability or induction of oxidative stress and inflammatory mediators (Wörle-Knirsch et al. 2007; Zhang et al. 2000, 2003).

Tungsten carbide (WC) nanoparticles are now being considered for the manufacture of hard metals to achieve extreme hardness and wear resistance, and mixing with cobalt is thought to improve toughness and strength of the material. In the past, occupational exposure to Co-containing dust in production facilities, which generally falls in the 1–20 μm size range (Stefaniak et al. 2008), has been associated with bronchial asthma, fibrosing alveolitis, and lung cancer (Lison 1996; Moulin et al. 1998). Tungsten carbide–Co (WC-Co) hard metal is now classified by the International Agency for Research on Cancer (IARC) as probably carcinogenic to humans, based on limited evidence in humans and sufficient evidence in experimental animals (IARC 2006). Experimental work has shown a higher mutagenic potential of the WC-Co mixture compared with its individual components (van Goethem et al. 1997), a finding that has been attributed to increased production of ROS. WC-Co exposure of peripheral blood mononucleated cells has been shown to trigger apoptosis of these cells via a caspase-9-–dependent pathway (Lombaert et al. 2004) and to generally up-regulate apoptotic and stress/defense response pathways (Lombaert et al. 2008). Ultrastructural analysis revealed that WC particles are incorporated into numerous vacuoles, whereas WC-Co particles lead to lysis of the cells, and no structural alterations due to Co particles could be demonstrated (Lison and Lauwerys 1990). Extensive studies on genotoxicity and mutagenicity have been conducted after a series of epidemiologic studies showed that hard-metal workers exposed to airborne WC and Co dust in occupational settings have increased mortality from lung cancer (Moulin et al. 1998).

Little information exists regarding effects of nanoparticles on other potentially exposed organs (i.e., skin, intestine, and the nervous system), although systemic circulation and distribution of inhaled or injected nanoparticles to different organs have been reported (Kreyling et al. 2002; Nemmar et al. 2001, 2002; Takenaka et al. 2001). Nanoparticles may trans locate to the central nervous system via the olfactory nerve (Oberdörster et al. 2005). A few neurotoxicologic studies have shown that titanium dioxide nanoparticles accumulate in microglial cells, causing increased ROS production, mitochondrial swelling, and membrane disruption (Long et al. 2006). Pisanic et al. (2007) reported reduction of neurite outgrowth and formation of intercellular connections after exposure of neurons to iron oxide nanoparticles.

In this study, we evaluated acute toxicity of WC and WC-Co nanoparticles in *in vitro* systems (i.e., human epithelial and rat neuronal and glial cells). Here we report the physicochemical characterization of WC and WC-Co nanoparticles in cell culture media and describe their intracellular distribution and cytotoxicity profile.

## Materials and Methods

### Particles

#### Preparation

**Figure 1:**
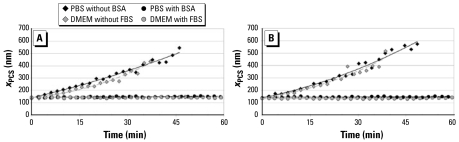
Effect of BSA in PBS and 5% (vol/vol) FBS in DMEM on the stability of the WC (*A*) and WC-Co (*B*) particles (10 μg/mL) compared to protein-free PBS and DMEM. We found identical results for WC and WC-Co in HBSS (data not shown).

WC and WC-Co particles (10% weight content of Co) were prepared by a chemical process and deaggregated and mixed, respectively, by means of a ball mill. We milled the nearly pure WC powder and Co powder in a hard-metal–lined ball mill using hard metal balls [see Supplemental Material, “Particle preparation and characterization,” and Supplementary Material, Table 1 and Figure 1 (http://www.ehponline.org/members/2008/0800121/suppl.pdf)].

From both types of particles, we prepared suspensions of 100 μg/mL in pure water (resistivity ≥ 18 MΩ.·cm; Wilhelm Werner GmbH, Leverkusen, Germany). For WC, water was sufficient to prepare electrostatically stable particle suspensions. For WC-Co, the addition of 0.01% (wt/vol) sodium poly-phosphate solution (Graham’s salt; CAS no. 10361-03-25; Merck, KGaA, Darmstadt, Germany) was necessary to obtain electrostatic stabilization of the particles, apparently due to the presence of Co. Graham’s salt is an often-used dispersant that is nontoxic in the applied concentrations. The suspensions were treated in an ultrasonic bath (RK 255 H; Bandelin, Berlin, Germany) for deagglomeration. After preparation, we quantified particle size and zeta potential. Time-dependent measurements in physiologic media (cell culture media or buffers) were performed by stirring a mixture of 90% (vol/vol) media and 10% (vol/vol) nanoparticle suspension in a beaker. The resulting suspensions were filled in a square cuvette for measurements. In parallel, studies were carried out in phosphate-buffered saline (PBS; Biochrom, Berlin, Germany) with or without bovine serum albumin (BSA; bovine fraction V, CAS no. 9048-46-8; Merck), which was dissolved in PBS before adding the particle suspension. We also performed experiments in Hank’s buffered salt solution (HBSS; Biochrom) or Dulbecco’s modified Eagle’s medium (DMEM; PAA Laboratories, Pasching, Austria) with or without 5% or 10% (vol/vol) fetal bovine serum (FBS; Invitrogen, Karlsruhe, Germany).

#### Physicochemical characterization

We determined the N_2_-BET specific surface area (BET; Brunauer, Emmet, Teller, after the developers of the basic calculations) of WC and WC-Co powders using an ASAP 2010 accelerated surface area and porosimetry analyzer (Micromeritics GmbH, Mönchengladbach, Germany). We determined the particle size distribution using dynamic light scattering (ZetaSizer Nano ZS; Malvern Instruments Ltd., Worcestershire, UK). We analyzed the mean particle size, *x*_PCS_, and the polydispersity index (PDI), which are described in DIN ISO 13321 (1996). We calculated the zeta potential from the Smoluchowski equation by measuring the electrophoretic mobility (ZetaSizer Nano ZS). These measurements were taken before and after autoclaving and yielded similar results. Therefore, we chose to sterilize the particle suspensions by autoclaving before exposure of cells. Solubility experiments were performed by centrifuging the nanoparticle suspensions at 15,000 × *g* (Sigma 4K15; Sigma Laborzentrifugen GmbH, Osterode am Harz, Germany). We then used clear supernatant to determine the tungsten and Co concentration using inductively coupled plasma–optical emission spectroscopy (Ultima; HORIBA Jobin Yvon, Unterhaching, Germany).

### Preparation of Co and tungsten salt solutions

We prepared cobalt chloride (CoCl_2_; Fluka/Sigma-Aldrich, Seelze, Germany) and sodium tungstate dihydrate solutions (Sigma) in distilled water at concentrations of 10 mM or 20 mM. Stock solutions were sterilized by autoclaving. We obtained all concentrations used in cell assays by serial dilution of the stock solutions with cell culture grade water (PAA Laboratories) under sterile conditions. Solutions were stored at 4°C.

### Cell culture

#### Cell lines

We used the following cell lines: CaCo-2 human colon adenocarcinoma cells [HTB-37; American Type Culture Collection (ATCC), Rockville, MD, USA), HaCaT human keratinocyte cells (CLS Cell Lines Service, Eppelheim, Germany) (Boukamp et al. 1988), A549 human lung carcinoma cells (CCL-185; ATCC), and OLN-93 oligodendroglial precursor cells (provided by the Department of Neonatology, Charité, Berlin, Germany).

#### Primary neuronal and astroglial cell cultures

Neuronal cell cultures were prepared from cortices of Wistar rat fetuses on gestation day 18 according to Fedoroff and Richardson (1997). Detailed descriptions of the routine maintenance of all applied cell cultures is available in the Supplemental Material [“Cell culture and assessment of cell viability” (http://www.ehponline.org/members/2008/0800121/suppl.pdf)].

### Exposure of cells to particles

The CaCo-2, HaCaT, and A549 cells were plated at a density of 5 × 10^4^ cells/well in a final volume of 500 μL in 24-well plates (Techno Plastic Products AG, Trasadingen, Switzerland) and allowed to attach for 24 hr before addition of particle suspensions. We then added 50 μL of the respective dilution of the particle suspension to 450 μL complete cell culture medium to reach final concentrations of 7.5, 15, and 30 μg/mL for WC nanoparticles and 8.25, 16.5, and 33 μg/mL for WC-Co nanoparticles. The water used to prepare the particle suspension (with or without Graham’s salt) was included as a control (vehicle).

We exposed cells to nanoparticle-containing solutions or vehicle control for 1 hr to 3 days. For exposure of cells with CoCl_2_ or sodium tungstate dihydrate, we added a maximal volume of 10% (vol/vol) salt solution to cell culture medium to reach the desired final concentrations. For coexposure of CoCl_2_ and WC, we added 5% (vol/vol) CoCl_2_ and 5% (vol/vol) of WC suspension to the cell culture medium to reach the same final concentrations of CoCl_2_ and 15 μg/mL WC.

We plated OLN-93 cells (1 × 10^4^ cells/mL) and primary cells (5 × 10^4^ cells/mL) in 96-multiwell plates in a final volume of 100 μL (Greiner Bio-One, Frickenhausen, Germany) and allowed them to attach for at least 24 hr before adding particle suspension. We added particle dilutions as described above, and cells were exposed for 3 days.

### Electron microscopy

For scanning electron microscopy (SEM) of cells, harvested cells were fixed with 2% (vol/vol) glutaraldehyde (Serva, Heidelberg, Germany) at room temperature, postfixed with 1% (vol/vol) osmium tetroxide (Roth, Karlsruhe, Germany), dehydrated in a graded series of acetone [including a staining step with 1% (vol/vol) uranyl acetate], and embedded in epoxy resin according to Spurr (1969). To avoid interference of the uranyl acetate and osmium tetroxide during energy-dispersive X-ray spectroscopy (EDX), we did not stain respective samples with the heavy metals; these samples were fixed in 2% (vol/vol) glutaraldehyde, dehydrated, and embedded in epoxy resin as above. Samples were cut on a Leica EM UC6 ultramicrotome (Leica, Vienna, Austria), equipped with a diamond knife (Diatome, Biel, Switzerland), carbon coated, and analyzed using a Philips XL 30 ESEM (Philips, Eindhoven, Netherlands). For EDX analyses, we used an EDAX detecting unit and EDAX software (version 3.0; EDAX Inc., Mahwah, NJ, USA).

### Assessment of cell viability

#### Light microscopy

Before termination of exposure, we observed cells by light microscopy using an inverse microscope.

#### Assays for cell viability

We determined cell viability using fluorescent indicator dyes that measure cellular metabolic activity [AlamarBlue (Biosource, Nivelle, Belgium) and Cell Counting Kit 8 (CCK-8; Dojindo Laboratories, Kumamoto, Japan)] and cell membrane integrity [5-carboxyfluorescein diacetate, acetoxymethyl ester (CFDA-AM) (Molecular Probes, Eugene, OR, USA)]. We followed procedures described by Schirmer et al. (1997) for AlamarBlue/CFDA-AM and the supplier’s protocol for CCK-8. Details of the procedures are provided in the Supplementary Material [“Cell culture and assessment of cell viability” (http://www.ehponline.org/members/2008/0800121/suppl.pdf)].

### Statistics

Exposure experiments with cells were performed in quadruplicate wells in three independent tests. We analyzed statistical differences using one-way analysis of variance (ANOVA) followed by Dunnett’s posttest (treatments vs. control), Tukey’s posttest (treatment vs. treatment), or two-way ANOVA followed by Bonferroni posttest using GraphPad Prism software (GraphPad Prism version 4.00 for Windows; GraphPad Software, Inc., San Diego, CA, USA). We considered *p-*values < 0.05 to be statistically significant. Fluorescence/absorbance units obtained in the cell viability assays were converted to percent of control and are presented as mean ± SD.

## Results

### Particles

#### Initial characteristics

The obtained BET specific surface area was 6.9 m^2^/g for WC and 6.6 m^2^/g for WC-Co. We expected the similarity in BET because WC-Co consists of 90% weight WC powder and both were milled under equal conditions. The particle size distribution and the morphology also were nearly the same, which we confirmed by two independent methods: SEM and dynamic light scattering. The mean particle size, *x*_PCS_^,^ which formed the initial value for studying the time dependence of agglomeration, was 145 ± 5 nm in both cases. The PDIs of 0.2 indicated a rather broad size range. We calculated the particle size range to be between 50 and 300 nm [see Supplemental Material, Figure 2 (http://www.ehponline.org/members/2008/0800121/suppl.pdf)]. The measured zeta potentials were approximately −35 mV for WC in water and −50 mV for WC-Co in sodium polyphosphate stabilized suspension. The high absolute values of the zeta potentials ensure a stable suspension in both cases because of the strength of the electrostatic repulsion force. Table 1 summarizes the physical parameters of both materials.

#### Dispersion stability in physiologic media

WC and WC-Co particles undergo agglomeration after addition to a protein-free physiologic medium. We studied the influence of different physiologic media (PBS, HBSS, DMEM) on agglomeration but found no differences. Likewise, we found no significant difference in the agglomeration behavior between WC and WC-Co particles. The absolute value of the zeta potential decreased after addition of the particles to PBS, HBSS, or DMEM. Zeta potentials for WC and WC-Co were between −20 and −23 mV.

In contrast to protein-free media, experiments with the protein albumin (BSA) in PBS showed no agglomeration of WC and WC-Co particles. Measured zeta potentials of the BSA-coated particles were about −11 mV. Although this small absolute value stands for a low electrostatic repulsion force, a steric component of the protein appears to lead to a stabilizing effect. To go one step further, we performed these investigations in DMEM or HBSS with 5% or 10% (vol/vol) serum to have the same conditions as in the cell culture experiments. There, we achieved the same stabilization effect for both serum concentrations. As already described for WC/WC-Co in PBS with BSA, the zeta potential of the particles in DMEM or HBSS with FBS was −11 mV. Figure 1 shows examples of the behavior of the particles in DMEM and PBS.

We determined the tendency of the particles to dissolve after we allowed the stock suspensions to stand for 1 week. For WC, 6% of the tungsten dissolved. For WC-Co, 15% of the tungsten and 76% of the Co dissolved. This level of dissolution of Co^2+^ from WC-Co is similar to that reported by Lombaert et al. (2004) for microsized WC-Co.

### Uptake of particles by cells

**Figure 2:**
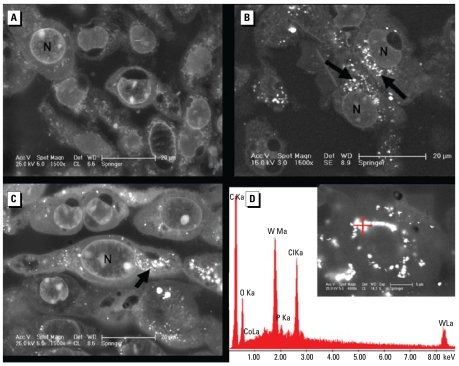
SEM (BSE) of embedded HaCaT cells after 2 days of incubation with medium without particles (*A*; control), 30 μg/mL WC nanoparticles (*B*), or 33 μg/mL WC-Co nanoparticles (*C*). Magnification, 1,500×. Heavy elements (e.g., tungsten and Co) appear as light areas (arrows in *B* and *C*), which are concentrated within the cytoplasm [gray regions around the nuclei (N)] but not inside the nuclei. (*D*) EDX analysis of the region indicated by a cross in the inset. We used the same conditions as for (*C*) (incubation with 33 μg/mL WC-Co particles for 2 days) but the sample was prepared without heavy metal staining. The spectrum shows two prominent X-ray peaks with the characteristic energy for X-ray quants originating from the W-M_α_ (W Ma) and W-L_α_ (W La) atomic shells, respectively. Other peaks represent further elements in the measured area and are due to compounds of the embedding media (ClKa, chloride *K*_a_; O Ka, oxygen *K*_a_), coating (C Ka, carbon *K*_a_), or compounds of the cell (P Ka, phosphorus *K*_a_; C, O). The Co-L_α_ (CoLa) peak of WC-Co is below the detection threshold.

To investigate whether WC or WC-Co nanoparticles are able to enter cells, we incubated HaCaT cells for 2 days with 30 μg/mL WC or 33 μg/mL WC-Co particles, respectively (Figure 2). Examination by SEM [back-scattered electron detector (BSE)] of epoxy-resin–embedded samples showed that particles and/or agglomerates with strong BSE signals were detectable in cells treated with WC particles (Figure 2B) and WC-Co particles (Figure 2C) but not in the control group (Figure 2A). Furthermore, the BSE signals of the particles/agglomerates are visible within the cells, but no agglomerates or particles were detectable inside the nucleus. Additional elemental analysis with EDX of one of the strong BSE signals caused by particles/agglomerates revealed an X-ray energy peak that exclusively belonged to tungsten, thereby indicating WC (or WC-Co; Figure 2D). These observations clearly confirm that nano-sized WC and WC-Co particles (or agglomerates) are able to enter cells. We likewise observed this for a series of other cells, including lung cells (A549) and oligodendrocytes (OLN-93; data not shown).

### Impact on cell viability

#### Particles

**Figure 3:**
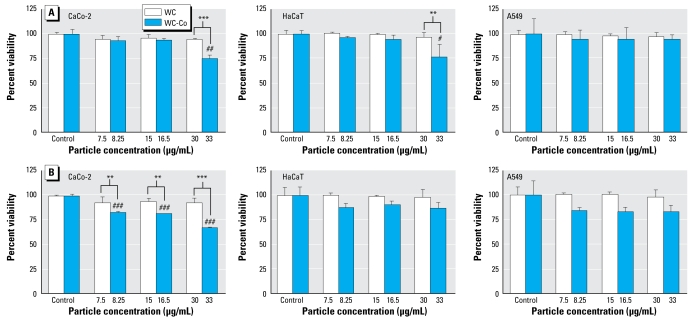
Cell viability, measured as metabolic activity with AlamarBlue (*A*) and as membrane integrity with CFDA-AM (*B*), of three human cell lines [CaCo-2 (left) HaCaT (center) or A549 (right)] after exposure for 3 days to WC-Co or WC. Results are expressed as a percentage of control cells that received identical treatment but without particles (mean ± SD; *n* = 3) ^#^*p* < 0.05, ^##^*p* < 0.01, and ^###^*p* <0.001 compared with control by one-way ANOVA with Dunnett’s posttest. **p < 0.01, and ***p < 0.001 by two-way ANOVA with Bonferroni posttest comparing both particle types. In (*B*), HaCaT cells showed overall treatment significance at *p* < 0.01 by two-way ANOVA.

**Figure 4:**
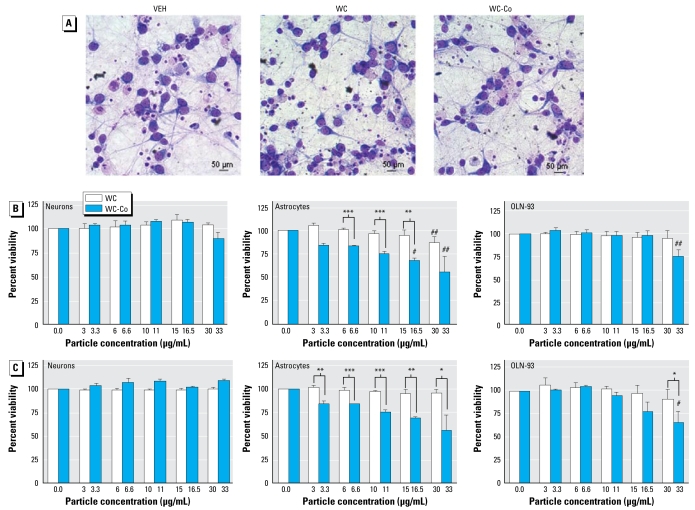
Effects of WC and WC-Co on cultured rat brain cells. (*A*) Light micrographs of neuronal cultures stained with Giemsa after a 72-hr exposure to vehicle (VEH; aqueous media used for particle preparations), WC particles (10 μg/mL), or WC-Co particles (11 μg/mL); there is no apparent difference in the appearance of the cultured neurons. (*B–C* ) Cell viability of primary neuronal (left) and astroglial (center) cultures and the oligodendroglial cell line OLN-93 (right) after exposure to WC and WC-Co particles over 3 days measured by the CCK-8 assay (*B*) or AlamarBlue (*C*). WC particles caused a small but significant effect on cell viability of astrocytes at the highest concentration; WC-Co particles significantly decreased viability in oligodendrocytes and astrocytes in a concentration-dependent manner. Two way ANOVA revealed that there was a significantly higher toxicity of WC-Co particles in astrocytes and OLN-93 cells compared with WC particles. Results are expressed as a percentage of control values (mean ± SD; *n* = 3–6) ^#^*p* < 0.05; ^##^*p* < 0.01; and ^###^*p* < 0.001 compared with control by one-way ANOVA with Dunnett’s posttest. *p < 0.05; **p < 0.01; and ***p < 0.001 comparing both particle types by two-way ANOVA with Boneferroni posttest.

WC nanoparticles did not yield a toxic response up to 30 μg/mL after 3 days of exposure in either of the human epithelial cell lines, the OLN-93 cell line, or the primary rat neuronal and astroglial cells (Figures 3 and 4). Exposure of the cell cultures to WC-Co elicited slight to substantial toxicity, with the order of sensitivity being primary astrocytes > intestinal cells (CaCo-2) > oligodendrocytes (OLN-93) > skin cells (HaCaT) > lung cells (A549) > primary neuronal cells (Figures 3 and 4). Comparison of the sensitivity of each cell line to WC and WC-Co particles over the tested concentration range by means of two-way ANOVA revealed that the addition of Co significantly increased toxicity of the particles for intestinal and skin epithelial cells as well as gliotoxicity (toxicity to astrocytes and oligodendrocytes).

#### Role of Co ions in toxicity

**Figure 5:**
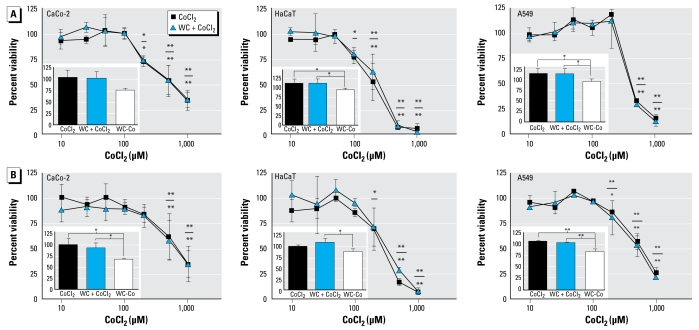
Cell viability (mean ± SD; *n* = 3), measured as metabolic activity with AlamarBlue (*A*) and as membrane integrity with CFDA-AM (*B*), of three human cell lines [CaCo-2 (left), HaCaT (center), and A549 (right)] is concentration-dependently decreased at ≥ 100 μM of CoCl2 after 3 days of exposure; addition of WC particles (15 μg/mL) did not alter these effects. Results are expressed as a percentage of control cells that received identical treatments but not Co or particles (*n* = 3). Insets show that WC-Co particles [33 μg/mL; maximum Co content of 51 μM (equal 10% wt/wt, or 3 μg/mL)] were significantly more potent in affecting cell viability than was CoCl_2_ (50 μM) or CoCl_2_ (50 μM) + WC (15 μg/mL). All cells were treated with the same total amount of Co. Cell viability is decreased in all cells that were exposed to WC-Co compared with cells exposed to either CoCl_2_ or WC + CoCl_2_ In (*A*),*p < 0.05; and **p < 0.01 compared with control by one-way ANOVA followed by Dunnett’s posttest. In (*B*),*p < 0.05; and **p < 0.01, between treatments by one-way ANOVA followed by Turkey’s posttest.

To investigate whether the Co fraction of WC-Co alone accounts for the toxic effects, we performed experiments using CoCl_2_. We found decreased viability of cells treated with CoCl_2_ starting from a concentration of 100 μM for the human cell lines (Figure 5). For comparison, the maximum Co concentration in the highest tested WC-Co particle concentration (33 μg/mL) is 51 μM. When cells were exposed to this Co concentration together with WC particles, we likewise did not observe toxicity (Figure 5, insets). We concluded that the Co fraction in the WC-Co particles alone could not account for the toxicity observed after treatment with WC-Co and that it is the combination of the particulate WC with Co that causes the cytotoxic effect (Figure 5, insets). We obtained similar results for the oligodendroglial cell line OLN-93 (data not shown).

#### Role of tungsten ions in toxicity

We tested tungsten salt (Na_2_WO_4_•2H_2_O) for its toxicity in OLN-93 cells and in astrocytes. We found no cytotoxicity of tungstate at the concentrations of 14, 28, and 42 μM, which correspond to 9%, 18%, and 28% dissolved tungsten. Cytotoxicity became apparent only at tungsten salt concentrations > 250 μM, which exceeds the level of tungsten present in any of the particle exposure experiments [for an example, see Supplemental Material, Figure 3 (http://www.ehponline.org/members/2008/0800121/suppl.pdf)].

## Discussion

Here we show that WC-Co nanoparticles can be toxic to mammalian epithelial, neuronal, and glial cells, whereas we detected no significant acute toxicity of WC nanoparticles in our test systems. Chemical–physical characterization confirmed that WC as well as WC-Co nanoparticles with an average diameter of 145 nm form a stable suspension in buffers that contain albumin and in cell culture media that contain serum. Cytotoxicity seen with WC-Co nano particles was higher than expected based on ionic Co content, indicating a qualitative cytotoxic effect of nano-sized particulate matter. Analysis by electron microscopy demonstrated the presence of WC nanoparticles within exposed mammalian cells.

Most of the chemical and physical parameters of WC and WC-Co nanoparticles are similar. Initial physical properties (*x*_PCS,_ PDI, BET) and the behavior in the physiologic media are identical. Both nanoparticles show similar agglomeration kinetics in all tested physiologic media. Furthermore, agglomeration of the nanoparticles can be inhibited in the presence of BSA or FBS. Obviously, the protein BSA adsorbs on the particle surface and stabilizes the suspension (Cedervall et al. 2007; Schulze et al. 2008). This result is comparable with stabilization of fullerene (C_60_) nanoparticles in PBS induced by human serum albumin (Deguchi et al. 2007). This stabilizing effect seems to apply to chemically different nanoparticles. Whereas fullerene is a special carbon modification, WC is a non oxide ceramic.

Ultrastructural analysis by electron microscopy revealed the presence of WC particles within exposed cells. The examination of the epoxyresin–embedded samples with different SEM methods (BSE detector signal, EDX analysis) enabled us to identify both the position and the chemical composition of the particles within the cells. The absence of the peak for Co in the EDX spectra may be explained by the low amount [only 10% (wt/wt) Co in WC-Co] and its tendency to dissolve. The lack of a Co peak may also be an example of possible artifacts that result from the extensive washing steps within the embedding procedure, which can reduce the EDX signal of Co below the detection threshold of the EDX detector. Nevertheless, the observations clearly confirm that nano-sized WC and WC-Co particles (or agglomerates thereof) are able to enter the cells. According to our findings, WC and WC-Co particles do not penetrate the nuclear membrane, because we detected no nanoparticles within the nuclei. It is, however, possible that particles with a size below the resolution of the SEM (therefore undetectable) can pass through the nuclear membrane. So far, we are not able to propose a mechanism for uptake of nano-sized WC or WC-Co particles or agglomerates based on the SEM and EDX data shown here. Endocytosis is a possibility. Stearns et al. (2001) documented this mechanism for uptake of ultrafine titanium dioxide particles by A549 lung cells. In contrast to the studies by Lison and Lauwerys (1990), who observed uptake of micro-sized (1–4 μm) WC and WC-Co particles by mouse peritoneal and rat alveolar macrophages, the mammalian cells used here and by Stearns et al. (2001) are not primarily phagocytic cells.

Nano-sized WC-Co particles, but not Co or tungsten ions at concentrations equivalent to the particulate form, or WC nanoparticles alone, led to acute toxicity in most of the cell cultures studied. The fact that the enhanced toxicity occurred only for WC-Co nanoparticles and not for WC particles with added Co^2+^ underlines the role of the particulate form for the expression of toxicity. An enhanced cytotoxicity of WC-Co was first reported by Lison and Lauwerys (1990) based on micro-sized dust in macrophage cells and was likewise shown to depend on the particulate form of the WC-Co mixture (Lison and Lauwerys 1992). In light of the genotoxic and carcinogenic potential of Co-containing hard metal particles and free Co^2+^ ions (Beyersmann and Hartwig 2008; Lison et al. 2001), and the current efforts to improve material designs based on WC-Co nanostructures, further studies are warranted to investigate whether nano-level WC-Co particles elicit size-specific toxic effects.

The mechanism behind the increase in toxicity for nano-sized WC-Co particles is not yet understood, but experiments with hard metal alloys suggested that the association of Co and carbide particles represents a specific toxic entity that produces large amounts of ROS. When both materials in particulate form are in close contact, electrons are transferred from Co to the surface of WC. This process leads to the production of activated (reduced) oxygen, and subsequently, oxidized Co (Co^2+^) passes into solution (Gries and Prakash 2007; Lison et al. 1995). The additional toxic effect of WC-Co may then be due to a combination of two known mechanisms of Co toxicity: cell damage induced by ROS derived from metallic Co (Hoet et al. 2002), and subsequent inhibition of DNA repair mechanisms by the Co^2+^ ions generated in this process (Kasten et al. 1997). In the rat, acute lung toxicity of a mixture of WC and Co was more pronounced than toxicity of individual components. The amount of excreted Co was higher in WC-Co–exposed animals compared with an equivalent amount of Co. This may be due to an increased bio-availability of Co from WC-Co (Lasfargues et al. 1992).

For nano-sized particles, however, an additional mechanism proposed by Limbach et al. (2007) may be relevant. They suggested a “Trojan horse”-like uptake of toxic ion with the metallic nanoparticle acting as carrier. In cases of toxic ions, the particle helps to cross the cell membrane barrier, thereby enhancing toxicity of the material because the damaging agent is delivered directly into the cell. This mechanism is likely to be involved with WC-Co–mediated toxicity, because a 3-fold higher Co uptake from the WC-Co mixture compared with Co metal powder alone could be demonstrated (Lison and Lauwerys 1990).

In the *in vitro* experiments, we observed that acute toxicity of WC-Co nanoparticles differed depending on the cell culture systems. Most sensitive were astrocytes and colon epithelial cells. Oligodendrocytes, human keratinocytes, and lung epithelial cells showed decreased viability at the highest concentration of WC-Co applied. Interestingly, neurons were resistant to acute application of WC or WC-Co nanoparticles. Little is known so far about the differential sensitivity of the cell lines, for example, with regard to oxidative stress. However, the CaCo-2 cell line appeared more vulnerable to oxidative stress and DNA damage due to a cyano bacterial toxin than were a human astrocytoma and a human B-lymphoblastoid cell line (Zegura et al. 2008). Differences in sensitivity of brain cells to oxidative stress have also been reported. Feeney et al. (2008) used organotypic hippocampal slices to study toxicity of hydrogen peroxide and found that astrocytes were more sensitive to oxidative stress than were neurons. In addition, Dringen et al. (2005) compared peroxide metabolism in cultured brain cells (astrocytes, oligodendrocytes, microglia, and neurons) and found that cultured oligodendrocytes disposed of the peroxide quicker than did the other neural and glial cell cultures. These findings suggest that different brain cells show variable efficiency in dealing with oxidative stress, with the observed susceptibilities conforming with the results of our study, which demonstrate that astrocytes are the most vulnerable brain cells to toxicity of WC-Co. These results indicate that vulnerability of different tissues to WC-Co nanoparticles will differ *in vivo*, as well. If WC-Co nanoparticles were to reach all organs to an equal extent, the highest toxicity would be expected within the nervous system because astrocytes were most vulnerable. Although neurons were not susceptible to acute toxicity of WC-Co nanoparticles, ongoing studies suggest that neurons react with increased production of ROS (data not shown). Thus, although we did not observe acute neuronal loss, it is possible that chronic exposure of neurons, which are nondividing, long-living cells, may eventually result in slow degeneration *in vivo*. Also, because astrocytes were very susceptible and because there is close interaction between astrocytes and neurons within the brain, secondary effects in neurons can be expected. Particulate matter has been shown to penetrate into the central nervous system of mammals and cause neuro degeneration (Campbell et al. 2005; Peters et al. 2006).

## Conclusions

Additional research is needed to evaluate mechanisms of acute toxicity of Co-doped WC nanoparticles in mammalian cells and evaluate biochemical pathways that account for differences in susceptibility of cell types. Our approach of using multiple cell cultures and cytotoxicity end points has proven valuable in revealing the combinatorial nanoparticle-mediated effects important for hazard assessment. An important next step is to take the exposure concentrations *in vitro* and *in vivo* into account to identify whether the potential target sites described by us are indeed prone to significant nanoparticle exposure. *In vivo* toxicology will thus help address whether systemic toxicity and neurotoxicity may occur after exposure to WC-Co.

## Figures and Tables

**Table 1:** Physical properties of investigated nanoparticles.

Substance	BET (m^2^/g)^a^	X_PCS_ (nm)^b^	PDI	Zeta potential (mV)
WC	6.9	145 ± 5	0.2	−35
WC-Co	6.6	145 ± 5	0.2	−50

a Specific surface area.

b Mean particle size.
